# Evidence for more cost-effective surveillance options for bovine spongiform encephalopathy (BSE) and scrapie in Great Britain

**DOI:** 10.2807/1560-7917.ES.2017.22.32.30594

**Published:** 2017-08-10

**Authors:** Ben A Wall, Mark E Arnold, Devi Radia, Will Gilbert, Angel Ortiz-Pelaez, Katharina DC Stärk, Ed Van Klink, Javier Guitian

**Affiliations:** 1 Royal Veterinary College, London, United Kingdom; 2 Animal and Plant Health Agency, Weybridge, United Kingdom; 3 University of Bristol, United Kingdom

**Keywords:** bovine spongiform encephalopathy, BSE, cost-effectiveness, economic analysis, epidemiology, policy, scrapie, surveillance, transmissible spongiform encephalopathies

## Abstract

Transmissible spongiform encephalopathies (TSEs) are an important public health concern. Since the emergence of bovine spongiform encephalopathy (BSE) during the 1980s and its link with human Creutzfeldt–Jakob disease, active surveillance has been a key element of the European Union’s TSE control strategy. Success of this strategy means that now, very few cases are detected compared with the number of animals tested. Refining surveillance strategies would enable resources to be redirected towards other public health priorities. Cost-effectiveness analysis was performed on several alternative strategies involving reducing the number of animals tested for BSE and scrapie in Great Britain and, for scrapie, varying the ratio of sheep sampled in the abattoir to fallen stock (which died on the farm). The most cost-effective strategy modelled for BSE involved reducing the proportion of fallen stock tested from 100% to 75%, producing a cost saving of ca GBP 700,000 per annum. If 50% of fallen stock were tested, a saving of ca GBP 1.4 million per annum could be achieved. However, these reductions are predicted to increase the period before surveillance can detect an outbreak. For scrapie, reducing the proportion of abattoir samples was the most cost-effective strategy modelled, with limited impact on surveillance effectiveness.

## Introduction

Bovine spongiform encephalopathy (BSE) is a prion disease of cattle, first identified in 1986 in Great Britain (GB) (GB refers to England, Wales and Scotland, whereas the United Kingdom (UK) also includes Northern Ireland. This study was based on GB-level data, however some of the data referred to were recorded at UK level). The epidemic in GB reached its peak in 1992 with ca 37,000 BSE cases [1], evolving into a public health crisis when it was linked to a human neurodegenerative condition, variant Creutzfeldt–Jakob disease (vCJD), in 1996. To date, a total of 229 deaths from vCJD have been recorded, of which 177 were in the UK [2,3]. However, a recent UK survey of human appendix tissue indicated that 493 (95% confidence interval (CI): 282–801) per million people are asymptomatic carriers of the abnormal vCJD prion [4]. Control measures and trade restrictions imposed during the BSE epidemic resulted in devastating economic consequences for the British beef industry. 

Scrapie is a prion disease that has been present in the sheep population in GB for several hundred years [5]. While there is no evidence linking scrapie to disease in humans, there have been concerns in the past that a scrapie outbreak could mask a hypothetical emergence of BSE in sheep. In addition, recent experimental studies using primate and humanised mouse models have indicated a zoonotic potential [6,7]. Cases of atypical scrapie and BSE have been detected in recent years, and their aetiologies remain unclear [1,8]. Atypical BSE occurs very rarely (only 10 cases have been detected to date in GB), while atypical scrapie appears to be endemic in GB.

A number of measures aimed at the eradication of these transmissible spongiform encephalopathies (TSEs) have been successful in that the incidence of both BSE and scrapie has fallen consistently in GB and across the European Union (EU) in recent years [1]. In order to determine the trend of TSE prevalence over time in EU Member States (MS), active surveillance is in place. This involves the testing for BSE in all EU MS of all cattle older than 48 months (24 months in some MS) which are either emergency-slaughtered for animal welfare reasons such as injury or illness or fallen stock which have died on the farm. Testing of healthy cattle slaughtered for human consumption was ceased in 2013 with certain exceptions (Bulgaria, Croatia and Romania) for the EU-25 [9]. Passive surveillance is also carried out via the notification of suspect clinical cases by veterinary surgeons. For scrapie, active surveillance, in the form of an annual survey of both fallen stock and healthy slaughtered animals, was added in 2001 and started in 2002 for all EU MS to the compulsory notification of suspect clinical cases, to monitor the incidence in sheep and the impact of disease control measures [10]. The presence of atypical disease forms is also monitored as a by-product of active surveillance, but this is not its primary aim. In recent years, the ratio of detected cases over the number of animals tested for both BSE and scrapie has been very low. In 2014 for example, ca 115,000 cattle were tested for BSE in GB, and just one case was detected [1]. There is therefore an economic incentive to re-evaluate current active surveillance systems to ensure that the resources deployed remain proportionate to the risk presented by a very low TSE prevalence. The re-allocation of funding could enable resources to be more effectively targeted at other diseases or issues, with beneficial outcomes for food safety. However, it is important that GB and the EU continue to have robust evidence to respond in a timely way to threats from new or re-emerging TSEs.

Previous studies have been conducted to evaluate BSE surveillance [11,12]. Most recently, to aid the determination of the level of sampling required, a model commissioned by the EU [13] demonstrated that a surveillance system based on the testing of at-risk animals (emergency slaughter, fallen stock and animals showing clinical signs) would be sufficient to detect a BSE case at a minimum prevalence of 1 in 100,000 in the standing population, with 95% confidence. However, this model was not able to explore the ability of such a surveillance system to detect re-emergence of birth cohort-based increases in BSE. For scrapie, models have focused on the estimation of prevalence of animals or holdings from surveillance data [14-16], rather than exploring the optimal design of a surveillance scheme.

The potential public health impact of TSEs and resulting political concern are important considerations in the formulation of surveillance strategies, and in situations where state or EU legislation apply, the research should be framed within this context. Animal health scientists have historically tended to focus on technical aspects of disease control without consideration of economic and socio-political impact [17]. Cost-effectiveness analysis allows the consideration of the financial value of a change, framed by a measure of the impact that change may have on society. The aim of the present study was to evaluate the cost-effectiveness of alternative surveillance strategies for BSE and scrapie, while explicitly considering the policy implications of these changes.

## Methods

The approach taken was firstly to simulate a number of BSE and scrapie surveillance scenarios and estimate the time taken to detect a statistically significant increasing trend in prevalence. Secondly, the total cost of surveillance between the start of the hypothetical increase and detection was calculated for each scenario. Thirdly, a number of measures for the technical assessment of surveillance were included in a cost-effectiveness analysis of the alternative scenarios. Finally, a number of interviews were conducted with policy experts and key stakeholders to discuss the results and the future of TSE surveillance. Each of these steps is described below in greater detail.

### Estimating time to detect significant increasing trend

The estimate of the prevalence in each birth cohort (BSE) or calendar year (scrapie) was obtained by means of the back-calculation approach used in Arnold and Wilesmith for BSE, and Arnold and Ortiz-Pelaez for scrapie [11,14]. Information on population size, test sensitivity and numbers tested, along with the assumed trend of infection each year, was used to generate the number of expected and the number of observed cases each year in GB for each surveillance activity. Variability was determined by Poisson distribution, which was used to simulate the observed number of cases from the expected values.

To estimate the time of detection, a model of the form *A*exp(*Bt*) was used (where *A* represents the infection prevalence at the start of the re-emergence, *B* represents the annual rate of increase, and *exp* denotes exponential growth), fitted to the infection prevalence in each birth cohort (BSE) or calendar year (scrapie), from the time when the prevalence started to increase. The first time point at which the rate of increase was statistically significant was recorded. The model was run 100 times for each scenario.

The scenarios to be modelled were defined during a stakeholder workshop involving policymakers from the Department for the Environment, Food and Rural Affairs (Defra), the Animal and Plant Health Agency (APHA) and the Food Standards Agency (FSA), UK government bodies which are concerned with food safety and/or livestock health. Scenarios modelled for BSE involved:

i) the proportion of fallen stock tested: 100% (baseline), 75% and 50%
ii) the hypothetical rate of increase in birth cohort prevalence: 40%, 20% and 10%
iii) the age of cattle tested: > 48 months (baseline), > 60 months and > 72 months
iv) the starting point: 2006 (relatively high prevalence) and 2013 (very low prevalence)

For scrapie, scenarios were based on:

i) the annual number of sheep tested: 20,000 (baseline), 15,000, 10,000 and 5,000
ii) the ratio of abattoir survey sheep versus fallen stock sheep tested: 33:76 (baseline), 100:0, 75:25, 25:75 and 0:100
iii) the hypothetical annual increase in standing population prevalence: 10% and 5%

At the same workshop, parameters to measure the technical effectiveness of surveillance were also decided by policy stakeholders. These were:

i the time to detection of an increasing trend in prevalence

ii) the number of infected animals (and sheep holdings for scrapie) in the standing population at the time of detection
iii) the number of infected animals presented at slaughter during the period from the start of the increasing trend to detection.

### Costing of core surveillance activities

The costs of TSE surveillance were considered from a public sector perspective. The approach was to calculate the variable cost of testing one fallen stock cow, one fallen stock sheep or one abattoir sheep in GB, taking into account the following surveillance activities: The cost of rapid testing (all animals), including: (i) sampling and administration costs at the sampling site, (ii) consumables for sampling, (iii) sending of samples by courier from the sampling site to the laboratory, and (iv) rapid testing at the laboratory. In the case of a positive or equivocal result on the rapid test, in addition: (i) transportation from the laboratory to the APHA and (ii) confirmatory testing. Fixed costs (such as sample archiving) were assumed not to change with respect to the number of animals tested and thus were not included in the analysis.

### Estimating cost-effectiveness

The costs of each surveillance scenario were obtained by stochastic simulation using @RISK 6 software [18]. The unit cost of testing one animal was multiplied by the number of animals tested per year and the number of years until detection of a statistically significant increasing trend in prevalence. To account for variability, a normal distribution was applied to the number of years until detection, using the mean and standard deviation (SD) values generated from the simulation model. There was variability in the cost of sample transportation, and this was incorporated into the model as a normal distribution using the mean and SD of quotes from several different couriers. The predicted number of detected cases, as well as the number of false-positive or equivocal results expected based on the specificity of the rapid test, were used to calculate the cost of confirmatory testing during the period. The simulation was run for 1,000 iterations. As costs over time were linear, the cost per annum was derived from the cumulative cost of each scenario.

The number of infected animals in the standing population at the time of detection was calculated using the simulated prevalence of infection at the time of detection and the most recent available UK cattle and sheep population data (from 2013) [19]. The number of infected sheep holdings in the standing population was estimated using UK data from 2010 [20].

In order to compare the cost-effectiveness of different surveillance scenarios, incremental cost effectiveness ratios (ICERs) were calculated as per Formula 1.

$$\mathrm{ICER}=\frac{-(C_{a}-C_{b})}{\;\;\;\;(O_{a}-O_{b})}\;/1,000$$

where *C* = cost; *O* = outcome; *a* = alternative surveillance scenario and *b* = baseline scenario. 

**Figure 1:**
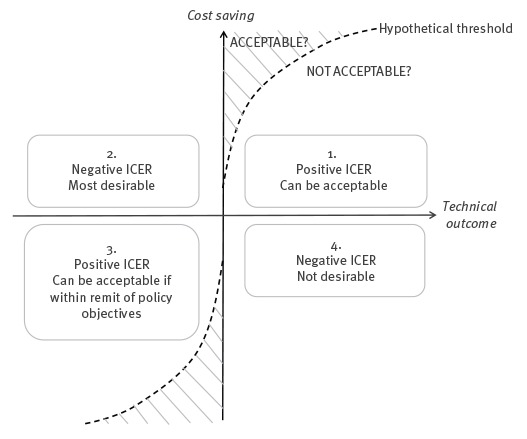
Key to interpretation of incremental cost effectiveness ratio plots

This provided an explicit expression of the difference in cost of an alternative scenario compared with the baseline, taking into account the difference in technical outcomes [21]. Using negative cost in this equation means that this ICER is a measure of money saved rather than money spent. ICER results are displayed in plots of costs saved against technical outcomes. Figure 1 shows a key to interpretation of ICER plots.

ICER: incremental cost effectiveness ratio.

ICERs with a positive value can fall within either quadrant 1 or 3. Surveillance scenarios in quadrant 1 can be acceptable in terms of cost-effectiveness, if they are within a threshold of acceptability (to be defined by policymakers). Scenarios with ICER values in quadrant 3 are likely to be unsuitable for policy objectives. A negative ICER value could indicate either the most desirable result (quadrant 2), or an undesirable outcome (quadrant 4), hence care should be taken in interpretation.

### Policy perspective

Finally, cost-effectiveness results were presented to policy stakeholders during a second workshop and telephone and email interviews were conducted with 15 experts in TSE science and policy from Defra, APHA, FSA, the European Food Safety Authority (EFSA), the European Commission, and higher education and research institutions. These semi-structured interviews explored stakeholders’ views on the purpose and future direction of surveillance, and the political landscape including trade and the public.

## Results

### Bovine spongiform encephalopathy

Table 1 summarises the simulation results for each scenario.

**Table 1:** Simulated technical outcomes (including 95% confidence intervals) and costs of scenarios for the surveillance of bovine spongiform encephalopathy, Great Britain

Scenario	Starting point	Rate of annual increase in prevalence (%)	% fallen stock tested (> 48 months)	Years until detection of an increase in prevalence	Infected animals presented at slaughter during period to detection	Infected animals in UK standing population at time of detection	Mean annual cost in million GBP
				Mean (SD)	Mean (SD)	Mean (SD)	
1	2006	40	100	7.61 (+/- 1.76)	111 (+/- 59)	260 (+/- 152)	2.86
2	2006	40	75	8.07 (+/- 1.79)	131 (+/- 80)	305 (+/- 194)	2.15
3	2006	40	50	8.68 (+/- 2.32)	162 (+/-126)	385 (+/- 305)	1.43
4	2006	20	100	8.37 (+/- 2.13)	67 (+/- 25)	74 (+/- 16)	2.87
5	2006	20	75	8.37 (+/- 2.39)	85 (+/ - 43)	126 (+/- 56)	2.14
6	2006	20	50	9.62 (+/- 3.14)	112 (+/- 71)	160 (+/- 92)	1.44
7	2006	10	100	8.36 (+/- 2.16)	66 (+/- 25)	74 (+/- 15)	2.86
8	2006	10	75	9.12 (+/- 2.85)	77 (+/- 36)	79 (+/- 22)	2.14
9	2006	10	50	10.57 (+/- 4.92)	95(+/- 67)	89 (+/- 33)	1.42
10	2013	40	100	14.97 (+/- 3.21)	88 (+/- 61)	70 (+/- 25)	2.87
11	2013	40	75	21.09 (+/ - 2.77)	116 (+/ -74)	64 (+/ - 2)	2.14
12	2013	40	50	22.40 (+/ - 2.62)	166 (+/ - 122)	53 (+/ - 27)	1.43
13	2013	20	100	21.35 (+/ - 3.61)	71 (+/ - 36)	66 (+/ - 44)	2.85
14	2013	20	75	22.76 (+/ - 3.57)	86 (+/ - 44)	86 (+/ - 58)	2.14
15	2013	20	50	26.09 (+/ - 3.92)	137 (+/ -78)	159 (+/ -103)	1.43
16	2013	10	100	32.42 (+/ - 9.25)	74 (+/ - 47)	39 (+/ - 32)	2.85
17	2013	10	75	36.59 (+/ - 5.72)	104 (+/ - 21)	55 (+/ - 30)	2.14
18	2013	10	50	42.23 (+/ - 5.25)	147 (+/ - 68)	93 (+/ - 47)	1.43
19	2006	40	100% > 60 months	8.58 (+/ -2.06)	153 (+/ - 103)	366 (+/ - 249)	2.43
20	2006	40	100% > 72 months	10.15 (+/ - 2.24)	261 (+/ - 189)	623 (+/ - 449)	2.03

SD: standard deviation; UK: United Kingdom.

Reducing the proportion of fallen stock tested resulted in considerable reductions in the annual cost of surveillance. However, it also had an impact on the effectiveness of surveillance, increasing the number of years until detection of an increasing trend in prevalence, the number of infected animals in the standing population at the time of detection and the number of infected animals presented at slaughter before detection (Table 1). The magnitude of this effect varied depending on the starting point and the rate of hypothetical increase in prevalence.

**Figure 2:**
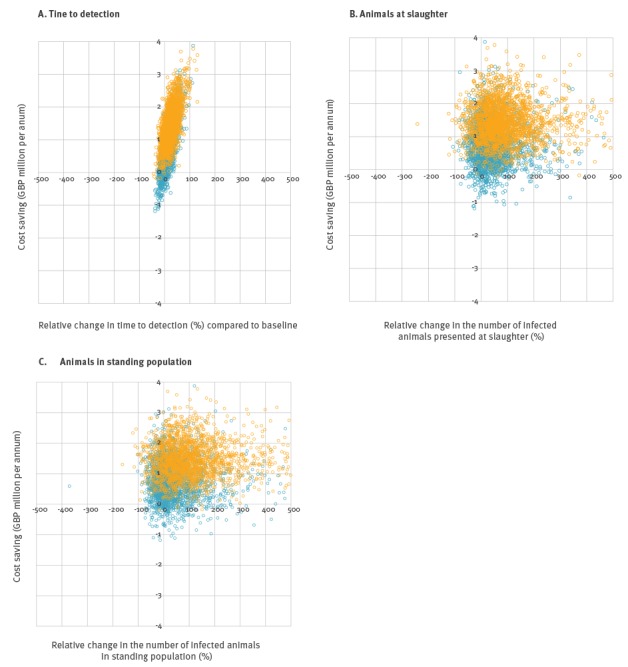
Incremental cost effectiveness ratios for alternative surveillance strategies for bovine spongiform encephalopathy, Great Britain

The ICER plots in Figure 2 show the distribution of the simulation results, derived from 1,000 iterations of the model. These plots demonstrated that strategies testing 75% or 50% of fallen stock may be considered acceptable in terms of cost-effectiveness, as their distributions fell mainly within quadrant 1. Strategies where 50% of fallen stock are tested provided a mean cost saving of GBP 1.43 million per annum, around twice the cost saving of strategies testing 75% of fallen stock (mean cost saving GBP 716,000 per annum). The distribution of values shown in Figure 2 reflects inherent variability and uncertainty in both the costs and technical outcomes, which were taken into account in the model.

ICER: incremental cost effectiveness ratio.

Blue dots: strategies where 75% of fallen stock are tested; orange dots: strategies where 50% of fallen stock are tested. The spread of points indicates the range, i.e. the uncertainty associated with the ICER, and is derived from 1,000 iterations of the model. For clarity, only scenarios involving the highest and the lowest starting point and rate of increase in prevalence are displayed (i.e. scenarios 2, 3, 17 and 18).

Mean ICER values for 75% and 50% testing strategies were, respectively: GBP 667,000 and 628,000 (saved per additional year to detection), GBP 40,000 and 28,000 (saved per additional infected animal presented at slaughter during the period to detection), and GBP 20,000 and 14,000 (saved per additional infected animal in the standing population at detection). The lower ICER values indicated that 50% testing strategies are less cost-effective in terms of every technical outcome modelled. However, given the establishment of a threshold of acceptability (as per Figure 1) they could potentially still be considered acceptable in terms of cost-effectiveness, and therefore adopted into policy, which would enable considerable cost savings.

Increasing the age at which fallen stock animals are tested performed less favourably than reducing the proportion of fallen stock tested in terms of cost-effectiveness (Table 1).

### Scrapie

Reducing the total number of sheep tested without changing the ratio of abattoir survey to fallen stock (i.e. scenarios 1–3 in Table 2) increased the time to detection of an increasing trend of scrapie prevalence by between 4.4% and 25.7%, or between 6.2% and 43.6% at a 10% or 5% rate of increase in prevalence, respectively (Table 2), along with similar proportionate increases in the number of infected animals and the number of infected holdings in the population at detection.

**Table 2:** Costs and simulated technical outcomes (including 95% confidence intervals) of various surveillance scenarios for classical scrapie, Great Britain

	Abattoir survey and fallen stock sampling scheme			Years to detection	Mean no. of infected animals presented at slaughter before detection	Mean no. of infected animals in standing population at detection	Mean no. of infected holdings at time of detection	Mean annual cost in GBP
	Scenario	Total no. of animals tested per year	AS:FS ratio	Mean (SD)				
10% annual rate of increase in prevalence	Baseline	20,000	33:67	11.19 (+/− 6.17)	2,100	29,411	712	565,637
	1	15,000	33:67	11.68 (+/− 7.30)	2,311	31,674	748	423,309
	2	10,000	33:67	12.58 (+/− 7.59)	2,631	36,198	819	285,738
	3	5,000	33:67	14.07 (+/− 8.61)	3,278	40,723	961	141,932
	4	20,000	100:0	11.73 (+/− 6.62)	2,266	31,674	748	793,899
	5	20,000	75:25	11.16 (+/− 7.22)	2,122	31,674	748	705,796
	6	20,000	25:75	10.41(+/− 6.90)	1,914	29,411	641	546,633
	7	20,000	0:100	10.84 (+/− 6.48)	2,029	29,411	677	466,698
5% annual rate of increase in prevalence	Baseline	20,000	33:67	16.18 (+/− 12.73)	2,594	22,624	534	568,968
	1	15,000	33:67	17.19 (+/− 14.14)	2,875	24,886	570	425,387
	2	10,000	33:67	17.74 (+/− 15.55)	3,082	24,886	570	282,690
	3	5,000	33:67	23.23 (+/− 17.32)	4,719	33,936	783	141,369
	4	20,000	100:0	15.98 (+/− 12.23)	2,501	22,624	534	798,568
	5	20,000	75:25	17.13 (+/− 11.74)	2,744	24,886	534	709,465
	6	20,000	25:75	15.98 (+/ − 12.38)	2,532	22,624	534	546,842
	7	20,000	0:100	16.06 (+/− 10.00)	2,494	22,624	499	460,393

AS: abattoir survey; FS: fallen stock; SD: standard deviation.

**Figure 3:**
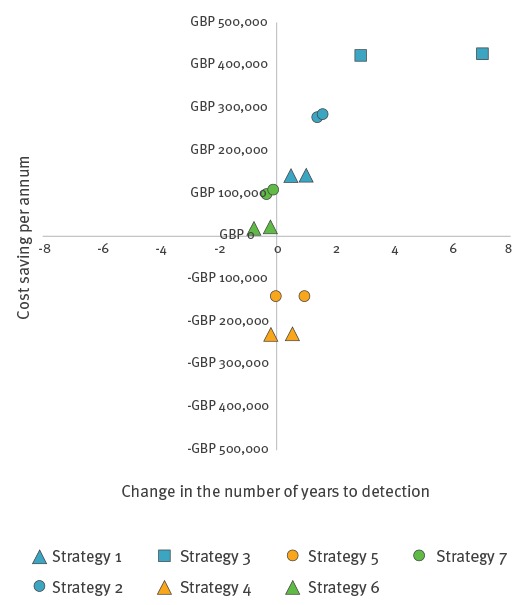
Incremental cost effectiveness ratio values for alternative scrapie surveillance scenarios, in relation to the number of years until detection of an increasing trend, Great Britain

**Figure 4:**
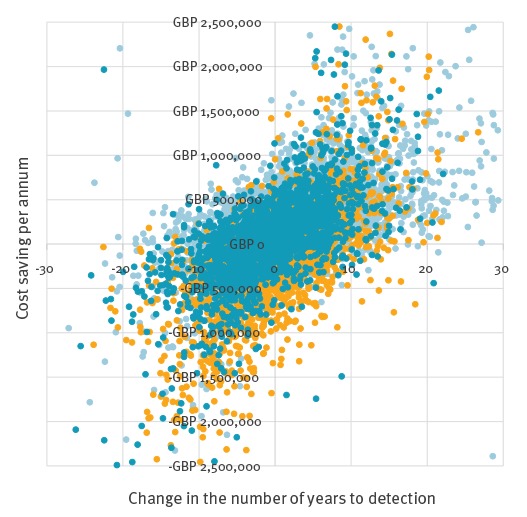
Distributions of incremental cost effectiveness ratio values for alternative scrapie surveillance scenarios, in relation to the number of years until detection of an increasing trend, Great Britain

When the mean model outputs for these strategies were plotted, they all fell within quadrant 1 (Figure 3), indicating that they can be considered to be cost-effective relative to the baseline depending on the threshold of acceptability. The mean ICERs for one of the technical outcomes (the number of years to detection) are shown in Figure 3 as an example. Figure 4 demonstrates the variation associated with these mean values, showing the distribution of results of multiple iterations of the model.

Strategies 1–3: changing the total number of animals sampled; strategies 4–5: increasing the ratio of abattoir survey to fallen stock tested; strategies 6–7: reducing the ratio of abattoir survey to fallen stock tested. For each strategy, scenarios involving 10% and 5% hypothetical rates of increase in prevalence are plotted.

Blue dots: scenarios 1–3; orange dots: scenarios 4–5; purple dots: scenarios 6–7. Only 500 iterations of the model are shown, for clarity.

Table 2 shows that changing the proportion of abattoir survey animals to fallen stock (scenarios 4–7) had a small influence on the mean number of years to detection as well as the other technical outcomes, but had a considerable impact on cost. The mean ICER values for all three technical outcomes (an example is shown in Figure 3) indicate that strategies which involve reducing the proportion of sheep tested in the abattoir survey (strategies 6 and 7) were the most cost-effective of all strategies modelled, as they were all located within the second (most desirable) and first quadrants. However, the distribution of simulation results for one of the technical outcomes – the number of years to detection (Figure 4) – shows that there are a large range of possible outcomes (note the difference in scale between Figures 3 and 4), some of which fall into the third quadrant. Outcomes in the third quadrant are not desirable in the context of the policy question addressed by the present study.

For all scenarios, the starting prevalence in animals was 0.00044 (ca 7,400 infected sheep), so there was a three- to four-fold increase in the prevalence in animals at detection depending on the scenario. The prevalence of infected flocks before the increasing trend was 0.0064 (ca 460 flocks), so there was a 2.8- to 3.6-fold increase in the prevalence of infected flocks at detection, depending on the scenario.

A reduction in the rate of prevalence increase from 10% to 5% across scenarios caused an increase in the mean time to detection (Table 2). However, the final prevalence of infected sheep and holdings was on average lower for the 5% increase per annum compared with the 10% increase.

### Policy

Some of the 15 experts who were interviewed were cautious about the idea of reducing BSE and scrapie surveillance, expressing that active surveillance plays a role in building trust with other countries in order to strengthen trade. Nonetheless, there were indications of some interest in a change in active surveillance. It is most likely that a reduction in BSE surveillance would be considered only if and when the UK achieves ‘negligible risk’ status by EU and World Organisation for Animal Health (OIE) standards. There were mixed views among experts as to what would be the potential impact on trade (if any) of reducing scrapie surveillance, and there is already some policy support for changing the ratio of sheep tested in favour of fallen stock.

## Discussion

Surveillance for BSE across the EU has been evaluated in terms of its ability to detect a test-positive animal at a minimum prevalence of 1 in 100,000 in the standing population with 95% confidence [22], and on this basis recommendations were made that healthy slaughtered cattle no longer need to be tested. While the calculations were rigorous, the choice of the target minimum prevalence is debatable and represents a balance between having a robust surveillance system and what is practical in terms of cost. Our study builds on this work firstly by providing a more thorough evaluation of the ability of a surveillance system to detect re-emergence, and secondly by linking the surveillance measures to cost. While this study uses data from GB, the model could easily be applied to data from other European countries in future analyses.

The model for BSE surveillance is dependent on assumptions regarding the age of onset, which was estimated using data from the original BSE outbreak [23] but could feasibly have changed in more recent cases. Analysis of animals born after the reinforced feed ban (BARBs) in GB (in July 1996) has shown that the age of onset has not changed significantly for BARBs compared with animals born before the total feed ban [24], although there are few BSE-infected BARB animals (150 in GB) compared with pre-BARB BSE cases (approximately 180,000 in GB). A lengthening of the incubation period, for example because of a lower average infectious dose, would make the time to detection longer than that predicted in the present study, although the incubation period is not sensitive to small changes in dose [25].

The approach for modelling scrapie surveillance was based on assumptions of an annual increase in scrapie prevalence in the standing population, whereas the approach used for BSE in this study was based on an increase in successive birth cohorts. For scrapie, estimation of prevalence in birth cohorts is problematic because the age of the tested animals is not recorded. The present model only considered an increase in the prevalence of classical scrapie, as that was deemed of much greater relevance to policy than an increase in atypical scrapie. The model framework developed in the present study could be adapted for use for atypical scrapie, although there are likely to be difficulties in estimating some of the necessary parameters, particularly the sensitivity of the rapid test. We found that if the proportion of sheep tested in the abattoir stream were reduced, there would be a reduction in the cost of surveillance while maintaining technical outcomes. However, EU approval would currently be required for such a change.

In addition to modelling the technical outcomes, the present study also links these with costs in the form of the ICER. The costs of each scenario were calculated for the hypothetical outbreak period only (i.e. from the start of an increasing trend in prevalence until detection), even though surveillance is continuous, as this enabled comparison between scenarios. It is useful for policymakers to identify a threshold of acceptability for ICERs. Standardised ICER thresholds are established in human healthcare settings [26,27] and help policymakers to decide upon whether a proposed scheme is appropriately cost-effective or not. In this case, policymakers would need to consider at what point a compromise in technical effectiveness becomes unacceptable, and whether this varies according to costs saved.

A considerable proportion of the simulation results for scrapie were located in the third quadrant, in which technical outcomes are improved given an increase in costs. This is a result of random variation, arising from the variability and uncertainty incorporated into the model. Outcomes in the third quadrant may technically be considered cost-effective but are not desirable from a policy perspective, given that the objective of the present study was to explore ways to reduce the cost of surveillance. This highlights that the mean ICER values should be carefully considered within the context of the range of possible outcomes.

Before active surveillance was implemented, passive surveillance was useful for detecting BSE cases during the early stages of the epidemic in the UK. However, since the decline in BSE cases, passive surveillance has become less useful, as it depends greatly on the vigilance of farmers and veterinarians and their ability to recognise clinical signs, which will naturally decline with increasing time since an epidemic. It could be hypothesised that in the future, active surveillance may eventually be replaced with a different approach, for example risk-based surveillance, but such a change would still need to be consistent with the regulatory framework.

TSE surveillance is part of a package of control measures, including the restricted feed ban which prohibits feeding mammalian meat and bone meal to livestock and the removal of specified risk material in abattoirs [28]. Consumers would continue to be protected by these controls regardless of any change in surveillance strategy.

Two of the TSE experts interviewed suggested to raise the age of fallen stock tested for BSE from the current 48 months. However, the present study found this to be a considerably less cost-effective strategy than reducing the proportion of fallen stock tested. This highlights the value of studies such as this to provide a robust basis on which experts can form opinions. Ultimately, a strong base of scientific evidence should underpin any future changes to TSE policy.
